# DRD4 Mitigates Myocardial Ischemia/Reperfusion Injury in Association With PI3K/AKT Mediated Glucose Metabolism

**DOI:** 10.3389/fphar.2020.619426

**Published:** 2021-01-27

**Authors:** Xue-song Liu, Jing Zeng, Yu-xue Yang, Chun-lei Qi, Ting Xiong, Geng-ze Wu, Chun-yu Zeng, Da-xin Wang

**Affiliations:** ^1^ Department of Cardiology, The Second Xiangya Hospital of Central South University, Changsha, China; ^2^ Department of Cardiology, Daping Hospital, The Third Military Medical University, Chongqing, China; ^3^ Clinical Medical College, Yangzhou University, Yangzhou, China; ^4^ The Hospital Affiliated to Medical School of Yangzhou University (Taizhou people’s Hospital), Taizhou, China

**Keywords:** dopamine receptor D4, ischemia/reperfusion injury (heart), glucose transporter 4, PI3K/Akt pathway, apoptosis

## Abstract

Ischemia-reperfusion (I/R) could cause heart irreversible damage, which is tightly combined with glucose metabolism disorder. It is demonstrated that GLUT4 (glucose transporter 4) translocation is critical for glucose metabolism in the cardiomyocytes under I/R injury. Moreover, DRD4 (dopamine receptor D4) modulate glucose metabolism, and protect neurocytes from anoxia/reoxygenation (A/R) injury. Thus, DRD4 might regulate myocardial I/R injury in association with GLUT4-mediated glucose metabolism. However, the effects and mechanisms are largely unknown. In the present study, the effect of DRD4 in heart I/R injury were studied *ex vivo* and *in vitro*. For I/R injury *ex vivo*, DRD4 agonist (PD168077) was perfused by Langendorff system in the isolated rat heart. DRD4 activated by PD168077 improved cardiac function in the I/R-injured heart as determined by the left ventricular developed pressure (LVDP), *+dp/dt*, and left ventricular end diastolic pressure (LVEDP), and reduced heart damage evidenced by infarct size, the release of troponin T (TNT) and lactate dehydrogenase (LDH). DRD4 activation diminished I/R injury induced apoptosis and enhanced cell viability impaired by I/R injury in cardiomyocyte, showed by TUNEL staining, flow cytometer and CCK8 assay. Furthermore, DRD4 activation did not change total GULT4 protein expression level but increased the membrane GULT4 localization determined by western blot. In terms of mechanism, DRD4 activation increased pPI3K/p-AKT but not the total PI3K/AKT during anoxia/reoxygenation (A/R) injury *in vitro*. Interestingly, PI3K inhibitor, Wortmannin, blocked PI3K/AKT pathway and depleted the membrane GULT4, and further promoted apoptosis showed by TUNEL staining, flow cytometer, western blot of cleaved caspase 3, BAX and BCL2 expression. Thus, DRD4 activation exerted a protective effect against I/R injury by promoting GLUT4 translocation depended on PI3K/AKT pathway, which enhanced the ability of glucose uptake, and ultimately reduced the apoptosis in cardiomyocytes.

## Introduction

Ischemia-reperfusion (I/R) injury is a major problem after coronary revascularization by the percutaneous coronary intervention (PCI) (Fröhlich et al., 2013). Cardiomyocytes suffer severe damage attributed to energy metabolism disorder. In order to optimize cardiac energy metabolism and oxygen consumption, glycolysis becomes the main mechanism by using glucose as fuel instead of fatty acids (Ussher and Lopaschuk, 2008). In this process, limited ATPs production by glycolysis does not maintain the normal cardiac function and induces cells death (Depre et al., 1999; Harmancey et al., 2013). Mounting of evidences suggest that improvement of glucose metabolism in cardiomyocytes could increase the heart’s tolerance to I/R injury (Gandhi et al., 2008; Lucchinetti et al., 2011; Zhang et al., 2015), improve cardiac function recovery (Rowe et al., 2010), and promote cardiomyocytes survival (Apstein, 1998). Thus, glucose metabolism plays a critical role in cardiomyocytes survival during I/R injury. However, how to treat heart I/R injury by regulating glucose metabolism is incompletely elucidated.

Glucose intake depends on glucose transporters on the membrane of cardiomyocytes, mainly including glucose transporters 1 (GLUT1) and glucose transporters 4 (GLUT4) (Manchester et al., 1994). GLUT1 widely and stably expresses on the cell membrane and regulates basal glucose intake during fetal and early postnatal life (Montessuit and Lerch, 2013). However, GLUT4, the most abundant transporter in cardiomyocytes, could translocate from intracellular vesicles to the plasma membrane in response to the ischemia and hypoxia, which could increase glucose uptake (Slot et al., 1991; Sun et al., 1994; Young et al., 1997; Li et al., 2016). It is reported that GLUT4-deficiency depresses glucose utilization during ischemia and develops irreversible cardiac dysfunction associated with limited ATP production during I/R injury (Tian and Abel, 2001). It is suggested that increasing GLUT4 on the membrane could improve the ability to resist I/R injury in cardiomyocytes.

As we know, dopamine receptors are classified into the D1-like (DRD1, DRD5) and D2-like (DRD2, DRD3, DRD4) subtypes according to their structures and pharmacology (Beaulieu et al., 2015). It is reported that *DRD4* mRNA is found in the human heart (Cavallotti et al., 2010), but its function is largely unknown. It has been proved that DRD4 is associated with glucose metabolism. DRD4 deficient mice has lower glucose metabolism in the prefrontal cortex compared with WT mice at baseline, while activation of DRD4 could augment glucose uptake *in vivo* (Michaelides et al., 2010). Meanwhile, the DRD4 antagonist could effectively inhibit glucose transport in PC12 cells (Dwyer et al., 1999). Moreover, activation of DRD4 protects against A/R-induced cell death, while inhibition of DRD4 represents the opposite effect, but the mechanism is still unknown (Shimada et al., 2010). Thus, the regulation of DRD4 activity may be a reasonable pattern to regulate glucose metabolism and treat heart I/R injury. However, the mechanisms need further study.

Together, we hypothesize that activation of DRD4 might improve glucose metabolism by promoting GLUT4 translocation under I/R injury in heart. In the present study, both animal and cell trials were conducted, and the cardiac function and glucose metabolism were analyzed, which would provide new insight into the therapeutic target for I/R injury.

## Materials and Methods

### Animals and Cells Trail

Adult SD rats (250–300 g) were perfused by Langendorff system *ex vivo*. Details of the experiments were described previously (Gonon et al., 1998). Briefly, rats were anesthetized by pentobarbital (60 mg/kg). The heart was excised and perfused by a Langendorff apparatus at a constant pressure of 55 mmHg. The phosphate free Krebs-Henseleit buffer (Tian and Abel, 2001) was continuously gassed with 95% O_2_/5% CO_2_ (pH 7.4) and warmed by a heating bath. The heart temperature was continuously monitored and maintained at 37 ± 0.5°C. Ischemia was induced by the cessation of perfusion for 45 min then followed by reperfusion 60 min with PD168077 (TOCRIS, United States) or not, then LVEDP, LVDP, +*dp/dt* were harvested. The procedures for care and use of animals were approved by the Ethics Committee and Animal Care and Use Committee of Third Military Medical University. All applicable institutional and governmental regulations concerning the ethical use of animals were followed.

Adult mice cardiomyocytes (AMCs) and neonatal rat ventricular myocytes (NRVMs) were obtained as our previous work (Wang et al., 2010; Wang et al., 2017). For A/R model, NRVMs were obtained from neonatal rat hearts (<3 days) and grown in DMEM containing 10% FBS at 37°C in a humidified incubator with 95% atmosphere and 5% CO_2_. After starvation for 12 h, cells were transferred into a hypoxic incubator containing 95% N_2_, 5% CO_2_ for 4 h; for reoxygenation, cells were rapidly transferred into a normoxic incubator containing 95% O_2_ and 5% CO_2_ for gradient time-point reoxygenation after anoxia with fresh DMEM and fetal bovine serum (Supplementary Figure S1A), then 8 h was chosen for A/R model.

### Immunofluorescence

Frozen section of heart tissue and slides containing NRVMs or AMCs were rinsed with PBS, then blocked with 10% normal goat serum. Next, the sections were incubated with DRD4 primary antibody (Sigma, Germany, 1:100 dilution) or GLUT4 primary antibody (Abcam, United Kingdom, 1:100 dilution) for 12 h at 4°C, then incubated with appropriate secondary antibody (Thermo, United States) for 2 h at 37°C, next, the DAPI and (or) DiO perchlorate (Solarbio, China, 5 × 10^−6^ M) were added for 10 s at room temperature and (or) for 15 min at 37°C. The immunofluorescence staining was visualized using Olympus confocal microscope at DAPI, DiO, 488 and 546 nm emission.

### TTC Staining

To measure the infarct size, perfused rat hearts were frozen at −20°C for 30 min and cut into slices (5–6 slices/heart), then incubated in a sodium phosphate buffer containing 1% 2,3,5-triphenyl-tetrazolium chloride (Sigma, Germany) for 15 min to visualize the unstained infarct region. The infarct areas and whole ventricle areas were determined by planimetry with ImageJ. The infarct size was calculated as infarct area divided by ventricle area.

### Western Blot

For total protein, heart tissues and NRVMs were lyzed in radio immunoprecipitation assay (RIPA) buffer supplemented with protease inhibitor cocktail tablet and phosphatase inhibitor tablet (Roche, Germany). Protein content was measured according to the Pierce BCA protein assay kit (Sangon Biotech, China). For membranous protein, membrane protein in heart tissues and NRVMs were separated by the Mem-PER™ Plus Membrane Protein Extraction Kit (Thermo Fisher, United States) following the manufacturer’s protocol (Phuchareon et al., 2015). Then equal protein lysates were resolved electrophoretically on denaturing SDS-polyacrylamide gels, and transferred to 0.22 μm nitrocellulose membranes. After blocking in 5% milk in TBST, membranes were probed with the following primary antibodies: DRD4 (Sigma, Germany), cleaved caspase 3 (CST, United States), PMCA (CST, United States), GLUT4 (CST, United States), p-PI3K (CST, United States), PI3K (CST, United States), p-AKT (CST, United States), AKT (CST, United States), ATP1A1 (CST, United States) and GAPDH (Abcam, United Kingdom). The appropriate secondary antibody (LI-COR, United States) was used. The ATPase alpha-1 (ATP1A1) and plasma membrane-type Ca2^+^-ATPases (PMCA) as the loading control of membrane protein, while GAPDH as loading control of the total protein. The results were tested by Odyssey system and quantified by ImageJ.

### Enzyme Linked Immunosorbent Assay

The LDH ELISA kit (Abcam, United Kingdom) and TNT ELISA kit (Thermo, United States) were used for measuring the LDH and TNT in perfusion liquid according to the manufacturers’ instructions.

### TUNEL Staining

TUNEL staining was performed by a *in situ* cell death detection kit (Roche, Germany) as described previously (Khan et al., 2013). Paraffin section (4 µm) were mounted on slides or slides containing NRVMs were conducted following manufacturer’s instructions. TUNEL positive cells were detected by Olympus confocal microscope.

### Flow Cytometry

NRVMs exposed to DRD4 agonist (PD168077, 10^−5^ M) during A/R injury were examined by PE Annexin V Apoptosis Detection Kit I (BD, United States) described previously (Schwartzenberg et al., 2007). According to manufacturer’s instructions, cells were stained with PE Annexin V and 7-amino-actinomycin (7-AAD) to detect early apoptotic cells (PE Annexin V+/7-AAD-events) and late apoptotic cells (PE Annexin V+/7-AAD + events), which were determined by flow cytometry (BD, United States).

### Cell Viability Assessment

After A/R or not, NRVMs were exposed to PD168077 (10^−5^ M) or (and) Wortmannin (10^−8^ M) for another 24 h in fresh DMEM. Next, 10 μl CCK8 (Dojindo, Japan) solution was added to each well and the plate was incubated for additional 2 h. Absorbance readings at 450 nm were obtained using a spectrophotometer (Thermo, United States). 10^−8^ M Wortmannin was chosen because cell viability was insusceptible when the Wortmannin dose was below 10^–7^ independently (Supplementary Figure S1B).

### Glucose Transport Assay

The study was carried out according to the method described previously (Kaliman et al., 1995). Briefly, after A/R or not, NRVMs were incubated with PD168077 or (and) Wortmannin for 30 min, then glucose-free incubation was performed for 45 min in Krebs-Ringer phosphate buffer (Asano et al., 1992), next 100 μM 2-deoxy-D-[^3^H] glucose (0.5 ìCi/ml) was added. Transport was stopped 20 min later and measured by a liquid scintillation counter (Microbeta Trilux, Finland).

### Statistics

The data were expressed as mean ± SEM. Two-tailed Student *t* test for normally distributed data and Mann-Whitney nonparametric test for skewed data that deviate from normality were used to compare two conditions. One-way analysis of variance with Bonferroni *post hoc* test for normally distributed data and Kruskal-Wallis nonparametric test for skewed data were used to compare ≥3 means. Repeated measures ANOVA was used for repeated measures data. Differences with *p* < 0.05 were considered statistically significant.

## Results

### The Expression of DRD4 in Cardiomyocytes

**FIGURE 1:**
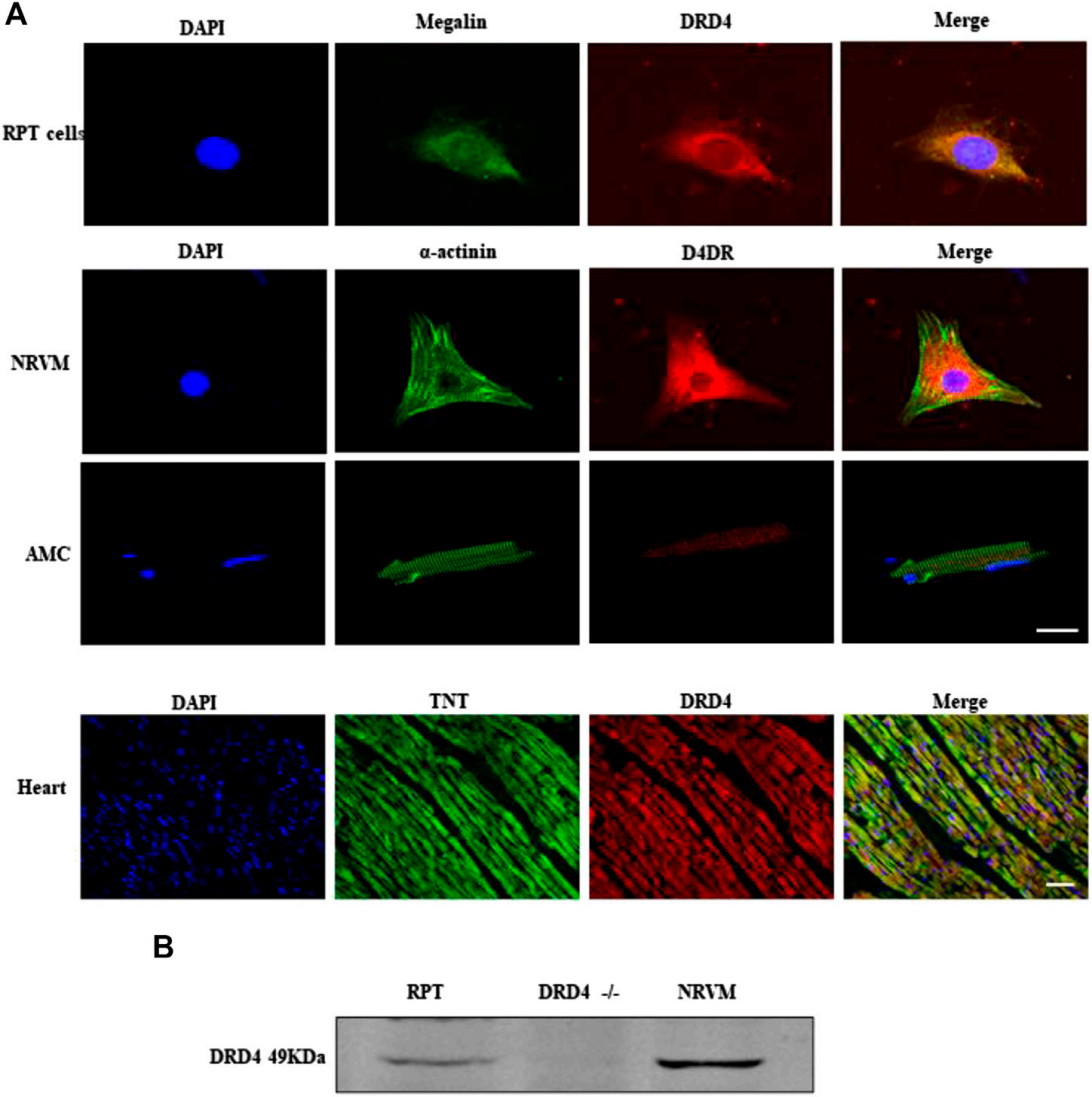
The expression of DRD4 in cardiomyocytes. **(A)** Immunofluorescence staining analysis DRD4 protein in renal proximal tubule (RPT) cells, neonatal rat ventricular myocytes (NRVMs), AMCs (adult mice cardiomyocytes, 8 weeks) and rat heart (8 weeks), Scale bar = 50 μm. **(B)** Western blot analysis of DRD4 protein in RPT cells, *DRD4* ^*−/−*^ mice and NRVMs.

Our previous works found DRD4 was expressed on kidney renal proximal tubule (RPT) cells (Chen et al., 2015; Zhang et al., 2016), so it was used as a positive control. As shown by immunofluorescence data (Figure 1A), the DRD4 was detectable in the RPT cells, NRVMs, AMCs and heart tissue. Meanwhile, this result was confirmed by western blot, which was observed that the DRD4 was expressed in RPT cells and WT rat heart but not the negative control, *DRD4* ^*−/−*^ mice (Figure 1B). These results indicated that DRD4 was expressed on rat and mice cardiomyocytes.

### DRD4 Activation Protects Heart From I/R Injury *Ex Vivo*

**FIGURE 2:**
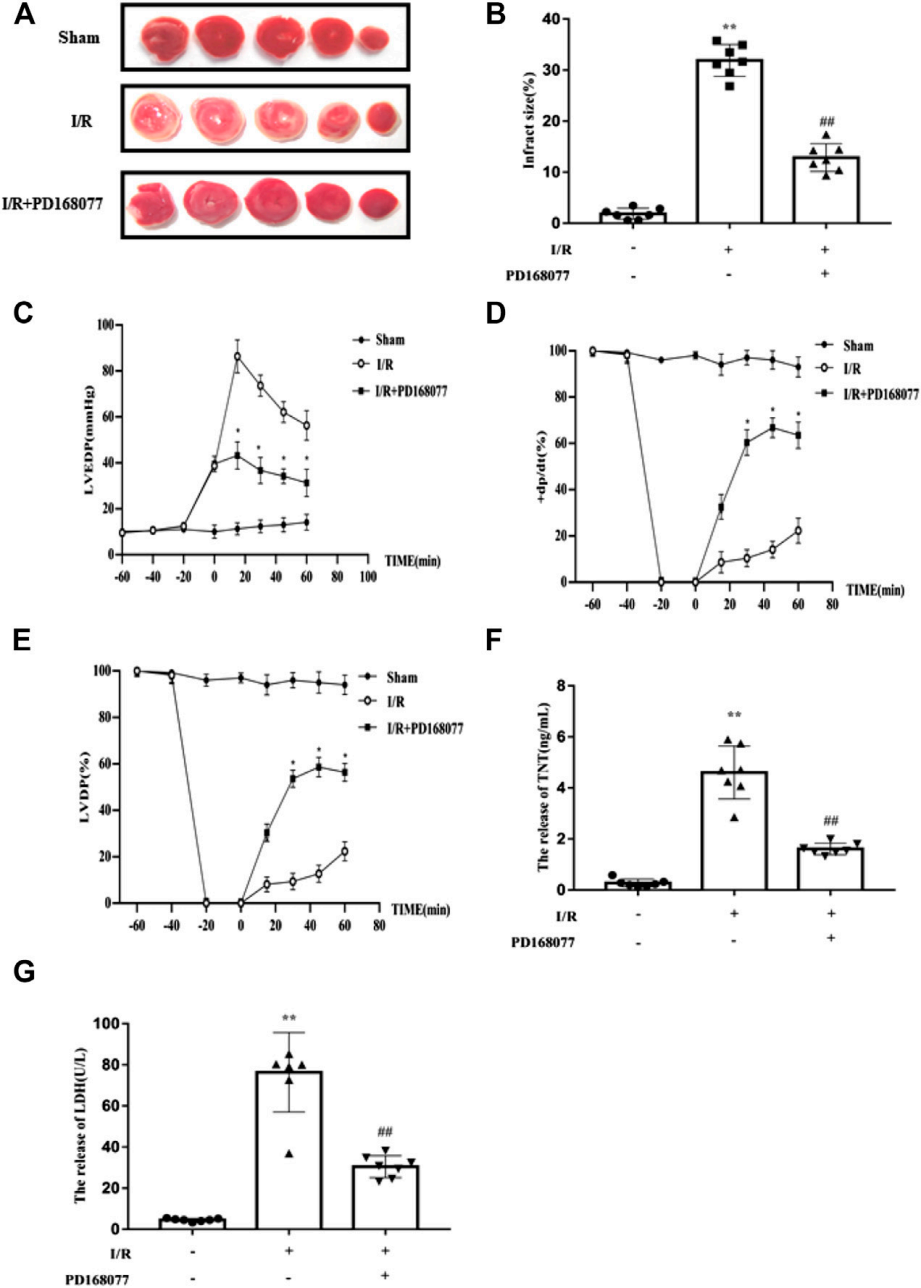
DRD4 activation protected the heart from I/R injury *ex vivo*. **(A)** TTC staining of rat heart after perfused by Langendorff system. **(B)** Quantitation of infraction size by ImageJ (*n* = 7, **p < 0.01 vs. Sham, ^##^ *p* < 0.01 vs. I/R). **(C–E)** The LVEDP, *+dp/dt* (%), and LVDP were measured by Langendorff system (*n* = 7, *p < 0.05, I/R vs. I/R + PD168077). **(F,G)** ELISA analysis of LDH and TNT in the fluid after perfusion (*n* = 7, **p < 0.01 vs. Sham, ^##^ *p* < 0.01 vs. I/R).

To analyze the role of DRD4 on cardiomyocytes during I/R injury, the isolated rat heart was perfused by Langendorff system. After that, the TTC staining was performed. Compared with the I/R group, DRD4 activation by PD168077 markedly decreased myocardial infarction size (Figures 2A,B), profoundly improved cardiac function, evidenced by the lower LVEDP (Figure 2C), higher *+dp/dt* (%) and LVDP (Figures 2D,E). Moreover, under PD168077 administration, the damage in cardiomyocytes was alleviated by decreased LDH and TNT in perfusion fluid compared with I/R group (Figures 2F,G). These results demonstrated that DRD4 activation improved cardiac function and decreased the damage in cardiomyocytes during I/R injury.

### DRD4 Activation Inhibit Apoptosis *Ex Vivo* and *In Vitro*

**FIGURE 3:**
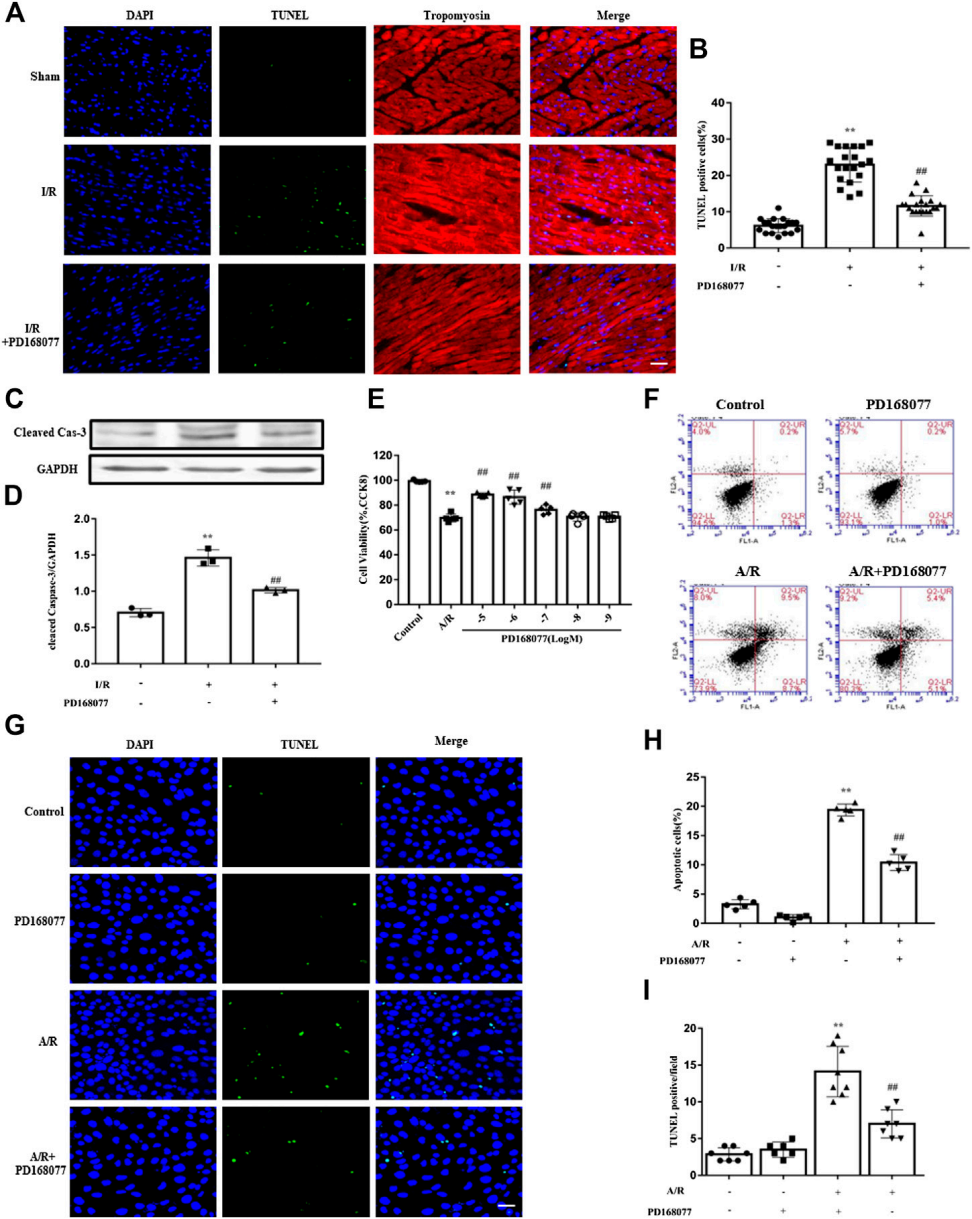
DRD4 activation decreased cardiomyocyte apoptosis and promoted cell viability. **(A,B)** TUNEL positive cardiomyocyte and quantification in heart section (*n* = 5, **p < 0.01 vs. Sham, ^##^ *p* < 0.01 vs. I/R, Scale bar = 50 μm). **(C,D)** Western blot analysis of cleaved caspase 3 protein in the heart (*n* = 3, **p < 0.01 vs. Sham, ^##^ *p* < 0.01 vs. I/R). **(E)** CCK8 assay of cell viability in different doses of PD168077 in NRVMs (*n* = 5, **p < 0.01 vs. Control, ^##^ *p* < 0.01 vs. A/R). **(F–I)** Flow cytometry and TUNEL staining of apoptosis, respectively, quantitated by flowjo and ImageJ, respectively. NRVMs were anoxic for 4 h, then reoxygenation for 8 h (*n* = 5, **p < 0.01 vs. Control, ^##^ *p* < 0.01 vs. A/R).

Cardiomyocytes are the main basis of cardiac function. Loss of them significantly worsen cardiac function. The TUNEL staining result showed that the apoptosis was enhanced under I/R injury, while this effect was diminished in DRD4 activation group (Figures 3A,B). Similarly, the expression of cleaved caspase 3, an apoptosis biomarker, was increased under I/R injury while reduced when DRD4 was activated by PD168077 (Figures 3C,D). Next, the effect of DRD4 on NRVMs was further determined. After A/R, the cell viability was blocked, while this effect was impaired by activation of DRD4 (PD168077, 10^−5^ to 10^−7^ M) (Figure 3E). Furthermore, the apoptosis cell was dramatically increased during A/R. However, this effect was mitigated after DRD4 activation (PD168077, 10^−5^ M) showed by flow cytometry (Figures 3F,H) and TUNEL staining (Figures 3G–I). These observations demonstrated that activation of DRD4 exert cardioprotection depended on decreasing apoptosis.

### DRD4 Activation Promote GLU4 Translocation *Ex Vivo* and *In Vitro*

**FIGURE 4:**
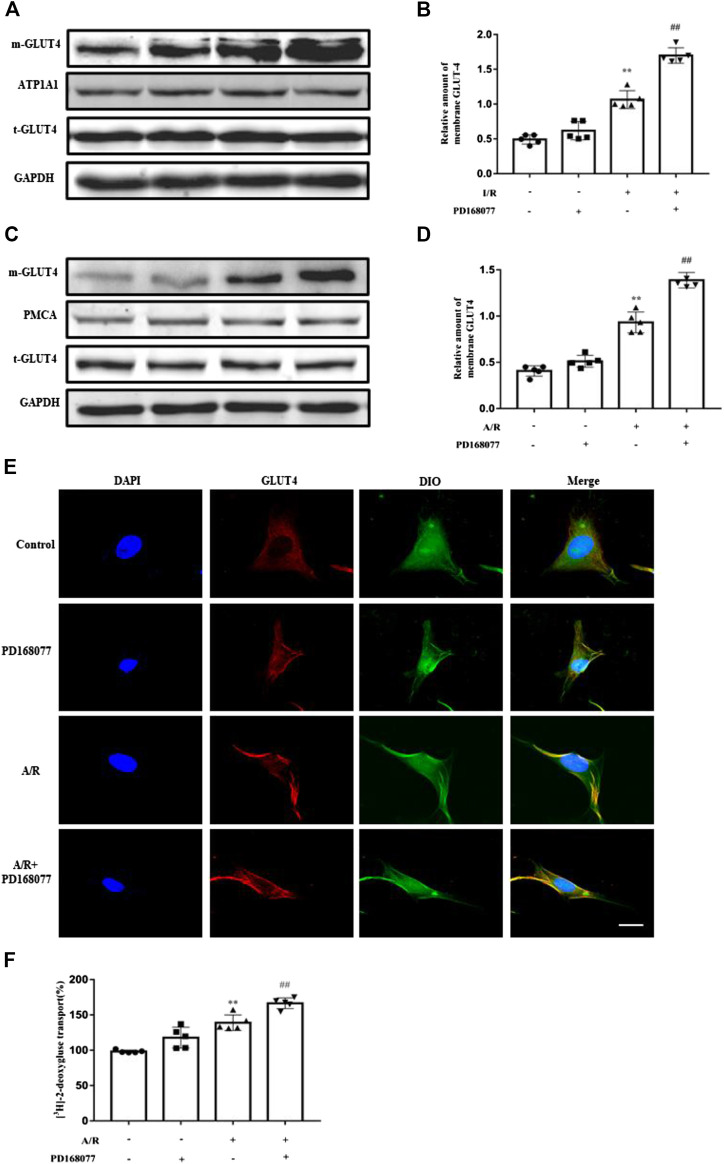
DRD4 activation promoted GLUT4 translocation from cytosol to membrane. **(A,B)** Western blot and quantitation of membrane GLUT4 protein in rat heart (*n* = 5, **p < 0.01 vs. Sham, ^##^ *p* < 0.01 vs. I/R). **(C,D)** Western blot and quantitation of membrane GLUT4 protein in NRVMs (*n* = 5, **p < 0.01 vs. Control, ^##^ *p* < 0.01 vs. A/R). **(E)** Immunofluorescence staining of GLUT4 translocation promoted by PD168077 in NRVMs, scale bar = 50 μm. **(F)** [^3^H]-2-deoxy-D-glucose uptake analysis in NRVMs. Data were collected as [^3^H]-2-deoxy-D-glucose uptake per microgram of protein, and results were expressed as fold change of the control (*n* = 5, **p < 0.01 vs. Control, ^##^ *p* < 0.01 vs. A/R).

Since glycolysis becomes the main energy source under I/R injury, glucose uptake becomes more critical, insufficient glucose supply induces apoptosis in cardiomyocytes. As we known, GULT4 is more prone to translocation due to the I/R injury. It was observed that the membrane GLUT4 was dramatically increased during the I/R injury, and this effect was enhanced after DRD4 activation in heart (Figures 4A,B), the similar results are also found in NRVMs after A/R (Figures 4C,D). Next, GLUT4 translocation promoted by PD168077 was confirmed in NRVMs by immunofluorescence staining, which was co-localized with the membrane-specific DiO (Figure 4E). Furthermore, increased GLUT4 could effectively promote glucose transport monitored by [^3^H]-2-deoxy-D-glucose uptake analysis *in vitro* (Figure 4F). These results suggested that activation of DRD4 reduced apoptosis through promoting GLUT4 translocation.

### Activation of DRD4 Reduces Apoptosis Through PI3K/AKT Mediated GLU4 Translocation *In Vitro*

**FIGURE 5:**
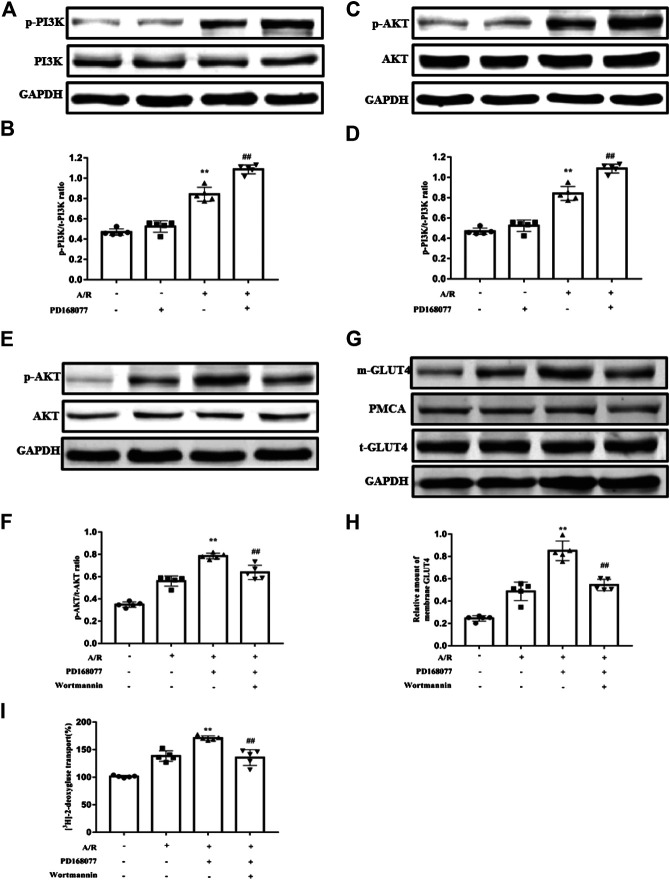
DRD4 activation promoted GLUT4 translocation *via* the PI3K/AKT pathway. **(A,B)** Western blot analysis of p-PI3K protein in NRVMs and quantitation. **(C,D)** Western blot analysis of p-AKT protein in NRVM and quantitation. **(E,F)** Western blot of p-AKT protein in NRVMs and quantitation. **(G,H)** Western blot of GLUT4 protein in NRVMs and quantitation. **(I)** [^3^H]-2-deoxy-D-glucose uptake analysis in NRVMs [**(A–I)** *n* = 5, **p < 0.01 vs. A/R, ^##^ *p* < 0.01 vs. A/R + PD168077].

As we know, GLUT4 translocation *via* the PI3K/AKT pathway was involved in I/R injury in cardiomyocytes (Matsui et al., 2001; Okumura et al., 2004; Cao et al., 2010; Ji et al., 2013; Chen et al., 2017). The current study found that p-PI3K and its downstream p-AKT were augmented during A/R injury, and this effect was enhanced by DRD4 activation but not the total PI3K or AKT (Figures 5A–D). Next, the PI3K inhibitor, Wortmannin, a covalent inhibitor of PI3Ks, was used to analyze the PI3K/AKT pathway does regulate the DRD4-induced GULT4 translocation. The p-AKT increase caused by activated DRD4 was weakened under wortmannin (10^−8^ M) treatment during A/R (Figures 5E,F), so did the GLUT4 on the membrane (Figures 5G,H). Moreover, increased GULT4 could effectively promoted glucose transport, while this effect was depleted by wortmannin, which was monitored by [^3^H]-2-deoxy-D-glucose uptake analysis (Figures 5I). These results demonstrated that activation of DRD4 induced GLUT4 translocation through the PI3K/AKT pathway.

**FIGURE 6:**
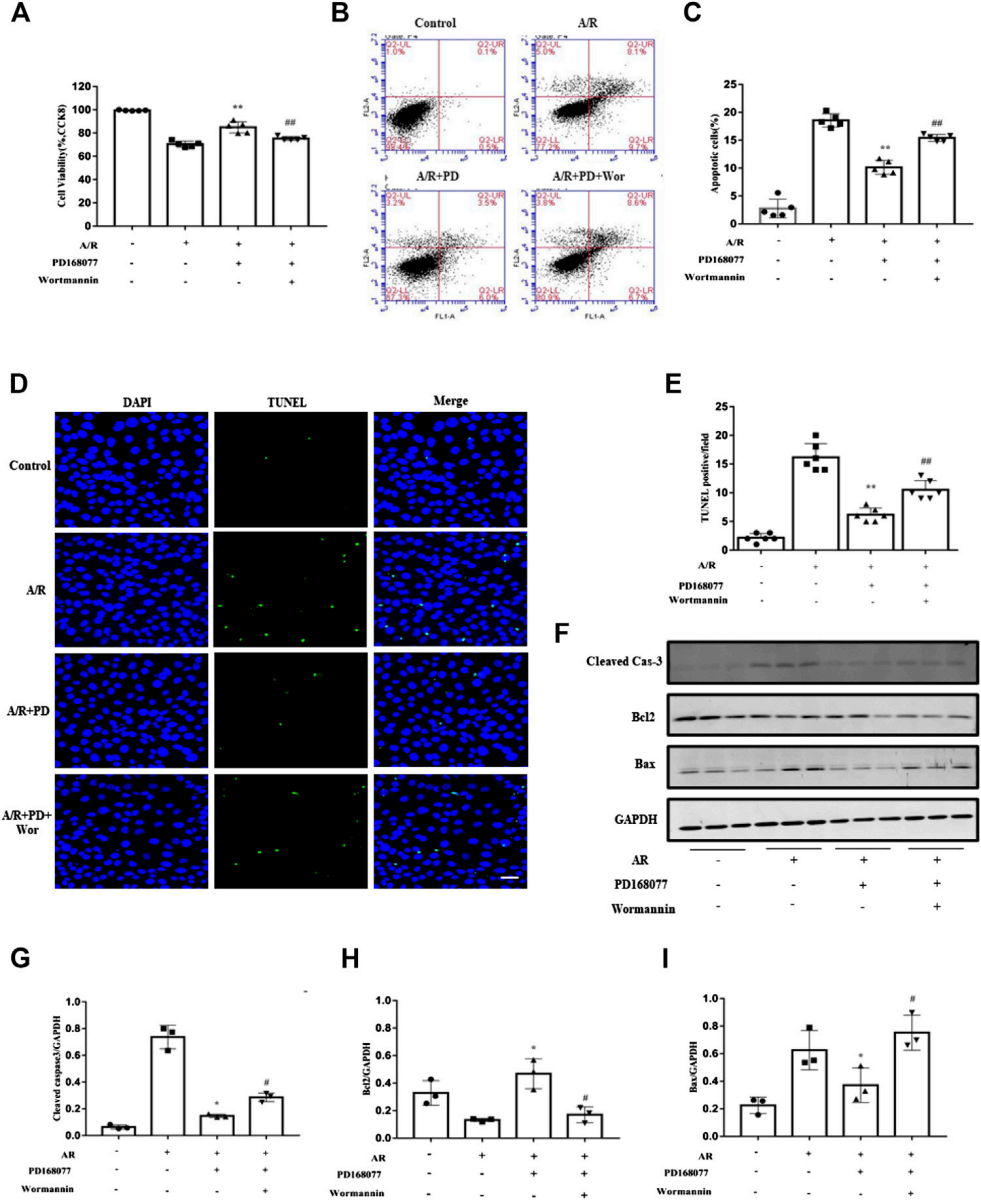
Inhibition of the PI3K/AKT pathway blocked the activated DRD4 mediated apoptosis in cardiomyocytes. **(A)** CCK8 assay of cell viability in NRVMs. **(B,C)** Flow cytometry and **(D,E)** TUNEL staining of apoptosis in NRVMs and quantitation, respectively [**(A–E)** *n* = 5–6, **p < 0.01 vs. A/R, ^##^ *p* < 0.01 vs. A/R + PD168077, Scale bar = 50 μm]. **(F–I)** Western blot of cleaved caspase 3, BCL2 and BAX protein in NRVMs after A/R injury and quantitation (*n* = 3, *p < 0.05 vs. A/R, ^#^ *p* < 0.05 vs. A/R + PD168077) PD, PD168077; Wor, wortmannin.

Whether blocking the PI3K/AKT pathway blocked the activated DRD4 mediated apoptosis in cardiomyocyte. Firstly, Wortmannin counteracted the increased cell viability which is enhanced by DRD4 activation (Figure 6A). Next, the role of Wortmannin about apoptosis was determined by Flow cytometry (Figures 6B,C) and TUNEL staining (Figures 6D,E), both results showed that DRD4 activation decreased apoptosis under A/R injury. However, this effect was offset by Wortmannin. Meanwhile, the effect of wortmannin on DRD4 mediated apoptosis was verified by the apoptosis marker, including cleaved caspase-3, BAX, and BCL2. Activation of DRD4 decreased cleaved caspse-3 and BAX, while increased BCL2 during A/R injury, however, this protective effect was offset by Wortmannin (Figures 6F–I). These data indicated that DRD4 activation reduced apoptosis through PI3K/AKT mediated GLU4 translocation *in vitro*.

## Discussion

The current study, for the first time, demonstrated that activation of DRD4 could improve the cardiac function, and reduce the apoptotic cardiomyocytes in I/R. Interestingly, DRD4 activation significantly improved glucose absorption by promoting GLUT4 translocation from cytoplasm to the cardiomyocyte membrane but not changing the total GLUT4 expression. Furthermore, activation of DRD4 activated a classic glucose metabolism pathway-PI3K/AKT pathway *ex vivo* and *in vitro*, showed by the higher p-PI3K and p-AKT protein expression levels (not the total- PI3K and total-AKT). On the contrary, inhibition of PI3K/AKT could reduce the GLUT4 translocation and enhance apoptosis under A/R *in vitro*. Therefore, DRD4 possessed the potential to mitigate myocardial I/R injury, which was associated with the PI3K/AKT pathway related GULT4 translocation.

DRD4 is one of D2-like dopamine receptors (Klein et al., 2019). It is reported that pre-treatment with DRD4 agonist greatly improves endothelial cell viability and decrease apoptosis (Wang et al., 2019). DRD4 agonist protects nerve cells from glutamate-induced apoptosis, while antagonist reverse this effect (Ishige et al., 2001). Moreover, the activated DRD4 could protect HT22 cells against the A/R injury, evidenced by decreased LDH, TNT, and apoptosis (Shimada et al., 2010). In the current study, we verified that the activation of DRD4 improved cardiac function *ex vivo*. Firstly, the expression of DRD4 in the cardiomyocytes was confirmed. Next, PD168077 was added when reperfusion by Langendorff system. DRD4 activation improved left ventricular systolic function and decreased infarction size during I/R injury. As we know, cardiomyocytes are the basis of cardiac function, and loss of them significantly impair cardiac function. Next move, we were wondering whether DRD4 activation improved cardiac function and relieved myocardial injury by reducing apoptosis. Apoptosis, a process of programmed cell death, is exacerbated during I/R injury, which could be determined by some indicators, such as TUNEL staining and cleaved caspase 3. Results showed that I/R injury aggravated cardiomyocytes apoptosis, however, this effect was abolished by DRD4 activation. Moreover, *in vitro,* apoptosis was augmented after A/R injury, and this effect was depleted by DRD4 activation in the insolated cardiomyocyte. These observations implied that DRD4 protected the heart against I/R injury might through inhibiting apoptosis.

We know that fatty acid metabolism is the primary energy source of cardiomyocytes under normal conditions. However, glycolysis becomes the main energy source under I/R injury, and the requirement of glucose for cardiac function becomes readily apparent for maintain contractile activity and survival of cardiomyocytes (Montessuit and Lerch, 2013). Cardiac function and survival of cardiomyocytes are improved by augmenting glucose metabolism during I/R (Jeremy et al., 1993; Cardaci et al., 2010; Huang et al., 2011). GLUT4, a kind of glucose transporter, is found in the heart (Govers, 2014), can translocate from the cytosol to the membrane under I/R injury, which enhance glucose uptake in cardiomyocytes (Liang et al., 2008). It is reported that an increase of glucose uptake by enhancing GLUT four transplant to the membrane could improve the ability for resisting I/R injury in cardiomyocyte (Huang et al., 2009; Lucchinetti et al., 2011). Besides, it is suggested that DRD4 is involved in brain glucose metabolism (Volkow et al., 2013). DRD4 deficient mice show lower glucose metabolism compared with *WT* mice, while activated DRD4 could augment glucose uptake (Dwyer et al., 1999; Michaelides et al., 2010). In the present study, GLUT4, transplanting to the membrane was increased during I/R injury *ex vivo* and *in vitro*, which consisted with the previous study. However, this effect became more after DRD4 activation. Besides, the function of the GLU4 transplanted to the membrane was valid evidenced by increased isotopic labeled glucose. These results demonstrated that DRD4 might increase glucose uptake by regulating GLUT4 localization, thereby increasing energy metabolism and protecting cardiomyocytes from apoptosis.

In terms of mechanism, PI3K/AKT pathway is the key position, which regulates GLU4 translocation under I/R injury (Okumura et al., 2004; Cao et al., 2010). Activated PI3K/AKT pathway enhance GLUT4 translocation in cardiomyocytes thereby improve cardiac function under I/R injury (Matsui et al., 2001; Chen et al., 2017). Besides, the PI3K inhibitor significantly reduces GLUT4 translocation, thus depresses ischemic preconditioning-induced cardioprotection (Ji et al., 2013). PEDF (pigment epithelium derived factor)-mediated GLUT4 translocation and glucose uptake increase in hypoxic cardiomyocytes are prevented by PI3K/AKT inhibitor (Yuan et al., 2019). Strikingly, activation of DRD4 also triggers this pathway, inhibition of DRD4 block PI3K/AKT expression in amphetamine-treated rats, revealing that DRD4 might regulate PI3K/AKT pathway (Hsieh et al., 2014). In the present study, the ratio of p-AKT/AKT and p-PI3K/PI3K were more remarkably by activated DRD4 during A/R injury than the A/R group *in vitro*, accompanying with enhanced GULT4 translocation, cell viability and decreased apoptosis. To verify that GULT4 translocation was dependent on the PI3K/AKT pathway. Wortmannin, a PI3K inhibitor, was used. Wortmannin depleted p-AKT, the downstream of PI3K, and offset active DRD4 induced GULT4 translocation. Moreover, wortmannin treatment also offset the protective effect of DRD4 on apoptosis and cell viability. These observations indicated that DRD4 activation reduced apoptosis in association with GLUT4 translocation through the PI3K/AKT pathway.

In summary, the DRD4 activation exerted a protective effect against I/R injury through promoting GLUT4 translocate in the membrane *via* PI3K/AKT signal pathway, thereby enhanced the ability of the cell glucose uptake, ultimately reduced the apoptosis. The findings might provide a novel evidence for the prevention and treatment of I/R injury by using DRD4 agonist.

## Data Availability Statement

The original contributions presented in the study are included in the article/Supplementary Material, further inquiries can be directed to the corresponding authors.

## Ethics Statement

The animal study was reviewed and approved by Ethics Committee and Animal Care and Use Committee of Third Military Medical University.

## Author Contributions

D-XW and C-YZ: designed and supervised the whole experiments. X-SL, JZ, and Y-XY: performed the experiments. C-LQ, TX, and G-ZW: provided help during experiments.

## Funding

This work was supported by grants from Jiangsu Provincial Science and Technology Support Program (Social development, No. BE2010697), “Six talent peaks project” in Jiangsu Province (2014-SWYY-052), Jiangsu Provincial “333 Engineering” (BE2010697), Foundation for PLA Young Scientists (15QNP059).

## Conflict of Interest

The authors declare that the research was conducted in the absence of any commercial or financial relationships that could be construed as a potential conflict of interest.
