# Supplementary material for: DRD4 Mitigates Myocardial Ischemia/Reperfusion Injury in Association With PI3K/AKT Mediated Glucose Metabolism
Source: Front Pharmacol. 2021 Jan 27;11:619426. doi: 10.3389/fphar.2020.619426 (PMC7873565; doi:10.3389/fphar.2020.619426)
Supplement: Supplementary file 1 [file image1.pdf]

Supplement

Figure S1

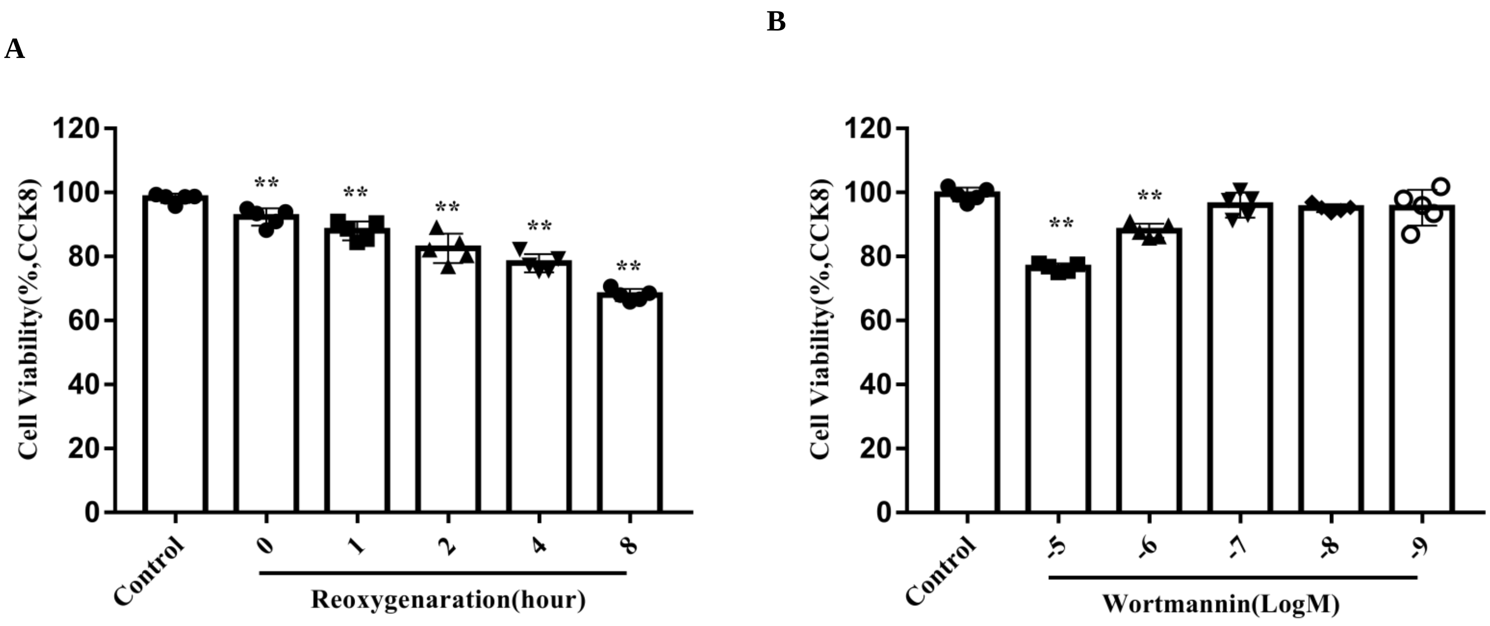

Figure S1 Cell viability with different time of reoxygenation **(A)** and different doses (10^{-9}M-10^{-5}M) of wortmannin. **(B)** in NRVMs. The data were evaluated by CCK8 assay. (n=5, * * P < 0.01 vs. Control)
